# Multiscale Interactome–Guided Prioritization of Candidate Herbs and Active Compounds for Hepatic Cirrhosis Using a Biased Random Walk Algorithm

**DOI:** 10.3390/cimb48030277

**Published:** 2026-03-04

**Authors:** Jun-ho Lee, Seon-Been Bak, Won-Yung Lee, Yun-Kyung Kim

**Affiliations:** 1 College of Herbal Medicine, Wonkwang University, Iksan 54538, Republic of Korea; 2 School of Korean Medicine, Dongguk University, Gyeongju 38066, Republic of Korea; 3 Department of Nutritional Science and Food Management, Ewha Womans University, Seoul 03760, Republic of Korea; 4 College of Korean Medicine, Wonkwang University, Iksan 54538, Republic of Korea; 5 Research Center of Traditional Korean Medicine, Wonkwang University, Iksan 54538, Republic of Korea; 6 Wonkwang Oriental Medicines Research Institute, Wonkwang University, Iksan 54538, Republic of Korea

**Keywords:** hepatic cirrhosis, multiscale network analysis, herbal candidates, active compounds

## Abstract

Hepatic cirrhosis is a progressive chronic liver disease driven by sustained inflammation, cell death, and tissue remodeling, and effective disease-modifying options remain limited. Here, we applied a multiscale interactome framework to prioritize candidate herbs and active compounds for hepatic cirrhosis. Herb–compound associations were collected from the OASIS database and mapped to experimentally supported compound–target interactions (DrugBank/TTD/STITCH), while cirrhosis-related proteins were curated from DisGeNET. Using a biased random-walk algorithm, we generated disease and herb/compound diffusion profiles on the multiscale network and ranked candidates by profile similarity and target overlap. Among the top-ranked herbs, Magnoliae Cortex, Notoginseng Radix et Rhizoma, Polygoni Cuspidati Rhizoma et Radix, and Capsici Fructus were supported by prior literature, whereas several high-ranking herbs lacked curated evidence and were highlighted as underexplored candidates, including Saposhnikoviae Radix and Fritillariae Cirrhosae Bulbus. Enrichment analyses indicated convergence on inflammatory and innate-immune pathways (TNF, Toll-like receptor, NF-κB) and apoptosis-related processes, with additional signals involving HIF-1 and PI3K–Akt pathways. Disease-focused subnetworks suggested mechanistic hypotheses for evidence-lacking compounds, including bergapten, oleic acid, and octadecanoic acid. Overall, we systematically prioritize herbal candidates and provides a mechanistic basis for follow-up validation in hepatic cirrhosis.

## 1. Introduction

Hepatic cirrhosis represents the end-stage of diverse chronic liver insults, characterized by extensive fibrotic replacement of functional parenchyma and architectural disruption that culminates in portal hypertension, synthetic failure, and life-threatening sequelae including ascites, variceal hemorrhage, hepatic encephalopathy, and jaundice. A 2019 global analysis estimated that hepatic cirrhosis together with other chronic liver diseases were responsible for roughly 1.47 million deaths worldwide, highlighting the formidable public-health burden of this condition [1]. The etiological spectrum spans chronic viral hepatitis (hepatitis B and C), excessive alcohol consumption, and metabolic dysfunction-associated steatotic liver disease; epidemiological data from the United States indicate that approximately 2.2 million adults carry a cirrhosis diagnosis, with age-adjusted mortality rates reaching 21.9 per 100,000 population [2]. Clinically, initial evaluation now favors noninvasive algorithms that combine serological fibrosis indices with transient or shear-wave elastography, followed by ongoing surveillance for decompensation once cirrhosis is confirmed [1]. Therapeutic strategies center on eliminating or controlling the causative insult and averting decompensation episodes, while liver transplantation remains the only curative option for advanced disease. Nevertheless, no approved disease-modifying antifibrotic therapy is currently available [3,4].

Given its inherently multi-component and multi-target pharmacology, herbal medicine has garnered increasing interest as a complementary therapeutic avenue for hepatic cirrhosis, a condition in which chronic inflammation and progressive fibrogenesis operate in concert and conventional management remains largely supportive [5]. Notable clinical evidence has emerged for specific herbal formulations: a multicenter, randomized, double-blind, placebo-controlled trial demonstrated that co-administration of Biejia-Ruangan with entecavir significantly enhanced fibrosis regression in chronic hepatitis B patients with advanced fibrosis or compensated cirrhosis compared with entecavir alone [6]. Similarly, a U.S.-based multicenter phase 2 trial showed that the botanical preparation Fuzheng Huayu was well tolerated and produced a statistically significant reduction in hepatic fibrosis among patients with chronic hepatitis C [7]. Taken together, these findings provide a strong rationale for a systematic, mechanism-oriented exploration of candidate herbs and bioactive constituents possessing disease-modifying potential in the context of hepatic cirrhosis.

Notwithstanding the encouraging clinical and preclinical evidence, the precise molecular mechanisms through which herbal medicines exert hepatoprotective and antifibrotic effects remain incompletely characterized, hampering evidence-based translation and rational drug design [8,9]. The emergence of systems biology and high-throughput omics technologies has propelled network pharmacology into a central role for delineating the complex relationships among herbal constituents, protein targets, and disease-associated pathways at a holistic level [10,11]. Such integrative pipelines have already proven valuable for prioritizing active ingredients and elucidating mechanistic pathways in various chronic diseases—for instance, elucidating the anti-diabetic mechanisms of Morus alba leaf extracts in type 2 diabetes mellitus and deconstructing the therapeutic actions of Epimedium herb in rheumatoid arthritis [12,13]. More recently, diffusion-based multiscale interactome frameworks employing biased random-walk algorithms have been developed to model how therapeutic agents propagate biological signals across hierarchically organized disease networks, thereby enabling systematic nomination of promising herbs and their active constituents [14].

**Figure 1:**
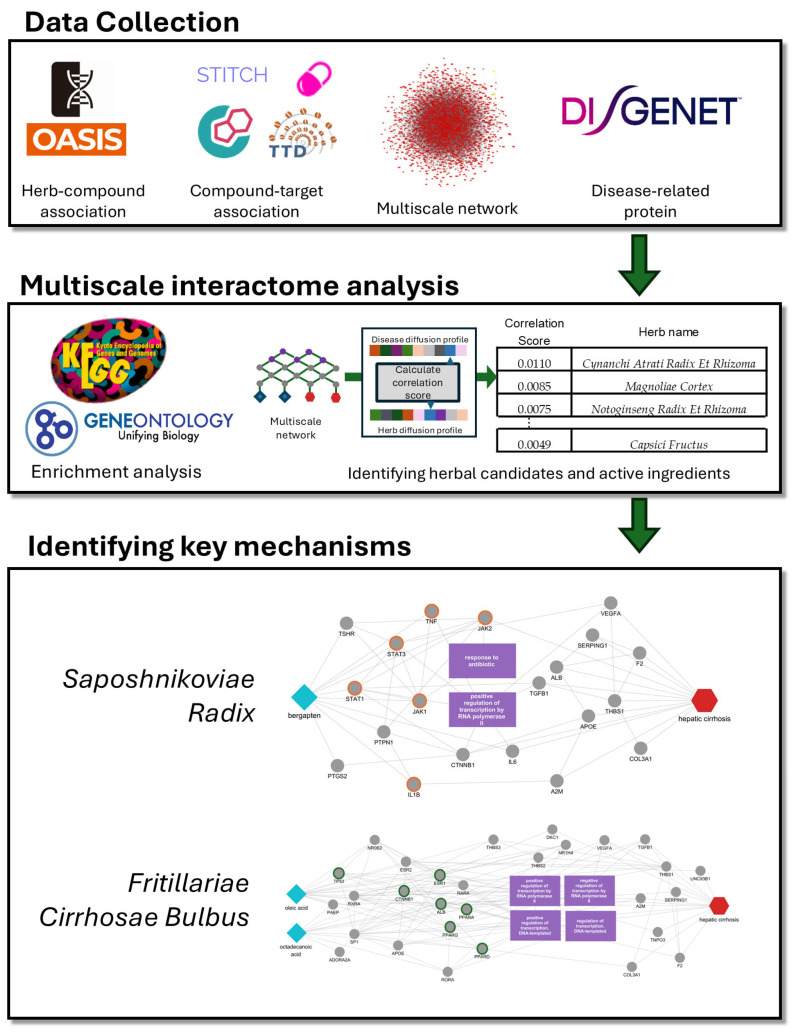
Overall workflow of the multiscale interactome–based framework for hepatic cirrhosis. Herb–compound associations were collected from OASIS and mapped to compound–target interactions using DrugBank/TTD/STITCH. Disease-related proteins were obtained from DisGeNET and integrated into a multiscale interactome. A biased random walk was used to generate diffusion profiles for the disease and each herb/compound, and candidates were prioritized by profile similarity (correlation) and target overlap. Enrichment analyses (KEGG/GO) and disease-focused subnetworks were then used to interpret key pathways and illustrate representative mechanisms for selected candidates.

In this study, we sought to systematically prioritize herbal candidates and their bioactive compounds with potential therapeutic relevance to hepatic cirrhosis and to delineate the underlying molecular mechanisms through a multiscale interactome-based network pharmacology framework. We began by assembling an integrated herb–compound–target knowledge base and curating a set of high-confidence cirrhosis-associated disease targets (Figure 1). These data were then projected onto a multiscale network encompassing protein–protein interactions, biological functions, and disease associations, and a biased random-walk algorithm was applied to generate diffusion profiles for both the disease and each candidate herb. Herbs were ranked by correlating their diffusion profiles with the hepatic cirrhosis disease profile, and those exhibiting high correlation scores and statistically significant target overlap were selected for further investigation. We subsequently characterized the shared protein targets and enriched biological pathways of the top-ranked herbs and extended the same diffusion-based analysis to individual constituent compounds to identify disease-relevant active ingredients and to propose mechanistic hypotheses linking them to cirrhosis-related pathological processes. Overall, this multiscale framework offers a principled, reproducible strategy for prioritizing multi-target herbal therapeutics and for connecting candidate herbs and their compounds to disease-relevant molecular pathways in hepatic cirrhosis.

## 2. Materials and Methods

### 2.1. Construction of the Herbal-Compound-Target Network

#### 2.1.1. Herbal Compound Dataset Construction

Herbal compound data were sourced from the OASIS platform (https://oasis.kiom.re.kr/index.jsp, accessed on 10 December 2024), a curated Korean medicine database maintained by the Korea Institute of Oriental Medicine (KIOM). Chemical constituent records were retrieved from the “Herbal Medicine–Physicochemical” module, yielding 16,117 distinct compounds across 478 herbal medicines. To ensure that each herb was represented by a pharmacologically meaningful number of constituents, herbs with five or fewer annotated compounds were excluded from downstream analyses.

#### 2.1.2. Construction of the Compound-Target Dataset

Experimentally validated compound–protein target interactions were compiled from multiple pharmacological databases, following the curation strategy described in a previous integrative interactome study [15]. Specifically, drug–target associations were gathered from DrugBank 5.0 [16]; the Therapeutic Target Database (TTD) contributed additional therapeutic target records, while the Search Tool for Interactions of Chemicals (STITCH) [17], supplied aggregated interaction data for over 430,000 chemicals derived from genomic, structural, and literature-mining evidence. Supplementary natural-product–protein interaction data, encompassing both direct and indirect binding events, were obtained from the dataset published by Huang et al. [18].

#### 2.1.3. Integration of Herbal-Compound and Compound-Target Data, and Network Construction

Herbal–compound associations from OASIS were merged with compound–target interaction records by matching PubChem Compound Identifiers (CIDs). After removing herbs with fewer than five mapped compounds, a tripartite network linking herbal medicines, chemical compounds, and protein targets was assembled. For each herb, compound–pathway connectivity was quantified by counting the number of enriched pathways per compound, and the top 50 protein targets per herb were retained for subsequent analyses. This filtering step ensured that the resulting network captured the most pharmacologically relevant interactions while maintaining a tractable graph size for downstream diffusion analysis.

### 2.2. Enrichment Analysis

Gene set enrichment analysis was carried out with Enrichr (http://amp.pharm.mssm.edu/Enrichr/, accessed on 22 December 2024) to identify signaling pathways and biological processes associated with the protein targets of candidate herbs and their constituents [19,20]. Enrichr queries multiple curated gene-set libraries—including Gene Ontology (GO) and the Kyoto Encyclopedia of Genes and Genomes (KEGG)—and reports adjusted *p*-values, z-scores, and combined scores for each enriched term. The combined score integrates the *p*-value and z-score through a logarithmic transformation, mitigating the individual limitations of each metric and providing a more robust ranking of enriched pathways. To control for multiple hypothesis testing, Benjamini–Hochberg false discovery rate (FDR) correction was applied across all enrichment tests, and only terms with an adjusted *p*-value (q-value) < 0.05 were considered statistically significant.

### 2.3. Disease-Gene Interaction Analysis and Protein Network Construction

Disease–gene associations for hepatic cirrhosis were obtained from the DisGeNET knowledge platform [21]. To maximize reliability, only expert-curated associations were retained; these were aggregated from UniProt, the Comparative Toxicogenomics Database, Orphanet, the Clinical Genome Resource (ClinGen), the Genomics England PanelApp, the Cancer Genome Interpreter (CGI), and the Psychiatric Disorders Gene Association Network (PsyGeNET). Associations derived exclusively from animal models or automated text-mining pipelines were excluded to minimize false positives, and gene–disease pairs annotated as “therapeutic” were likewise removed to focus on pathogenic mechanisms. The final gene set was further filtered to include only those genes whose protein products were represented in the multiscale interactome network (Section 2.4), thereby ensuring compatibility between the disease target space and the network topology.

### 2.4. Multiscale Interactome

The multiscale interactome adopted in this work was originally constructed by Ruiz et al. [22] and encodes three complementary layers of biological organization. The protein–protein interaction (PPI) layer comprises 387,626 physical interactions among 17,669 proteins, aggregated from the Biological General Repository for Interaction Datasets (BioGRID), the Database of Interacting Proteins (DIP), and the Human Reference Protein Interactome Mapping Project (HuRI). A protein–biological-function layer links 7993 proteins to 6387 Gene Ontology terms through 34,777 curated associations. Finally, a function–function layer organizes 9798 biological processes into a hierarchical network containing 22,545 parent–child relationships. This tripartite architecture enables the propagation of disease and therapeutic signals not only through direct molecular contacts but also through shared functional annotations, thereby capturing mechanisms that conventional single-layer networks may overlook.

### 2.5. Protein Overlap

A hypergeometric test was employed to assess whether the observed overlap between herb-associated protein targets and hepatic cirrhosis disease targets exceeded the expectation under a null model of random sampling. Formally, the *p*-value was computed from the hypergeometric distribution parameterized by: (i) *N*, the total number of proteins in the reference interactome; (ii) *K*, the number of hepatic cirrhosis–associated proteins; (iii) *n*, the number of herb-specific protein targets; and (iv) *k*, the number of proteins shared between the herb target set and the disease target set.

### 2.6. Diffusion Profile Calculation and Analysis

To quantify the mechanistic proximity between herbal candidates and hepatic cirrhosis at the network level, diffusion profiles were computed using a biased random walk with restart (RWR) on the multiscale interactome.

$$r^{\left(k+1\right)}=\left(1-\alpha \right)s+\alpha (r^{\left(k\right)}M+s\sum_{j\in J}r_{j}^{\left(k\right)})$$

The diffusion state at iteration *k* is denoted by $r^{\left(k\right)}$, where α represents the restart probability (i.e., the chance that the walker returns to the seed node set at each step) and s is the restart vector encoding the initial probability distribution over seed nodes. M denotes the biased transition matrix derived from the multiscale network, which weights edges according to their layer-specific topology. The restart probability was set to α = 0.85, following the parameterization adopted in the original multiscale interactome framework; this value balances local exploration around seed nodes with global network coverage.

$$||r^{\left(k+1\right)}-r^{\left(k\right)}|{|}_{1}>\varepsilon$$

The tolerance parameter, *ϵ*, was set according to previous studies. The correlation between the diffusion profiles of the drug and the disease was calculated using the following formula:

(1) $$\frac{\left(r^{\left(c\right)}-{\bar{r}}^{\left(c\right)}\right)\cdot \left(r^{\left(d\right)}-{\bar{r}}^{\left(d\right)}\right)}{||{(r}^{\left(c\right)}-{\bar{r}}^{\left(c\right)})|{|}_{2}||(r^{\left(d\right)}-{\bar{r}}^{\left(d\right)})|{|}_{2}}$$

$r^{\left(c\right)}$ and $r^{\left(d\right)}$ were used to represent the diffusion profiles of the component and the disease, respectively. The top k = 20 proteins or biological functions most heavily visited during the random walk were selected to construct disease-focused subnetworks. Alternative approaches for defining base functions in iterative mapping problems have also been explored [23]. Component targets that lacked connections to disease-associated proteins or biological functions within the subnetwork were excluded. Entities ranked highest in the diffusion profiles were interpreted as exerting the most substantial biological impact and were therefore prioritized for downstream mechanistic investigation.

### 2.7. Transcriptome Analysis

Transcriptome-level validation was performed using gene expression data retrieved from the HERB database (http://herb.ac.cn/, accessed on 7 January 2026) [24]. HERB aggregates experimentally derived transcriptomic profiles for a broad spectrum of bioactive compounds and maintains curated links to the Connectivity Map (CMap), enabling systematic comparison of compound-induced gene expression signatures. For each candidate compound, CMap-based similarity scores were extracted; compounds exhibiting a summary score ≥ 80 were classified as highly similar to the query compound, implying shared downstream transcriptional programs and potentially overlapping mechanisms of action. The functional activities of these highly similar compounds were then cross-referenced with hepatic cirrhosis–related literature to identify convergent pathways relevant to liver fibrosis and inflammation. In parallel, transcriptome profiles for selected target compounds were obtained from publicly available gene expression datasets, and differentially expressed genes (DEGs) were identified following standard preprocessing (background correction and quantile normalization). Genes satisfying the dual criteria of |log_2_(fold-change)| ≥ 1.0 and Benjamini–Hochberg adjusted *p*-value < 0.05 were retained as statistically significant DEGs. Gene Ontology (GO) enrichment analysis was subsequently performed using clusterProfiler to delineate the biological pathways and processes potentially modulated by these compounds in the context of hepatic cirrhosis.

### 2.8. Preparation of Herbal Extracts

Dried Fritillariae Cirrhosae Bulbus and dried Saposhnikoviae Radix were purchased from Human-Herb Co., Ltd. (Daegu, Republic of Korea). Each herbal material (50 g) was extracted with 70% (*v*/*v*) ethanol at a 1:10 (*w*/*v*) ratio using an ultrasonic bath (POWERSONIC 410, Hwashin Tech Co., Ltd., Seoul, Republic of Korea; 40 kHz). The extraction was performed in three 30 min cycles, while maintaining the bath temperature below 30 °C to minimize thermal degradation of heat-sensitive constituents. The filtrates from the three extraction cycles were combined and concentrated under reduced pressure at ≤30 °C (EYELA N-1110) and lyophilized (FDU-8606, Operon, Gimpo, Republic of Korea), yielding 1.90% and 11.83% (*w*/*w*) for the Fritillariae Cirrhosae Bulbus ethanol extract (FCB) and Saposhnikoviae Radix ethanol extract (SR), respectively. The dried extracts were stored at −80 °C. Immediately before in vitro assays, the extracts were dissolved in DMSO to prepare stock solutions and sterilized using a 0.22 µm PTFE syringe filter.

### 2.9. In Vitro Cytotoxicity and Anti-Fibrotic Activity Assay

LX-2 human hepatic stellate cells were seeded in 96-well plates and treated with water extracts of Saposhnikoviae Radix (SR) or Fritillariae Cirrhosae Bulbus (FCB) at 10, 30, 100, 300, and 1000 μg/mL for 24 h. For the fibrosis model, cells were co-treated with TGF-β1 (10 ng/mL) and each extract at the same concentrations. Cell viability was assessed by MTT assay (540 nm) and expressed as a percentage of the untreated control.

## 3. Results

### 3.1. Exploration of Potential Herbal Candidates for Hepatic Cirrhosis

To screen for herbs with potential therapeutic relevance to hepatic cirrhosis, herb–compound relationships were first compiled from the OASIS database and compound–protein target mappings were established through experimentally validated interaction resources (DrugBank, TTD, and STITCH). The assembled herb target sets, hepatic cirrhosis–associated disease targets, and the protein/biological-function nodes of the multiscale interactome were then used as inputs for a biased random-walk algorithm, which generated diffusion profiles for each herb and the disease. The degree of mechanistic proximity between a given herb and hepatic cirrhosis was quantified as the cosine correlation between their respective diffusion profiles. In parallel, a hypergeometric test was applied to evaluate whether the overlap between each herb’s protein targets and the disease target set was statistically significant.

Candidate herbs were retained only if they exhibited both a high diffusion-profile correlation score and a significant target overlap (adjusted *p* < 0.05 after Benjamini–Hochberg correction). From this filtered set, the top 10 herbs possessing at least three active compounds with significant disease-target associations were selected for further analysis. In descending order of correlation score, the prioritized herbs were Cynanchi Atrati Radix Et Rhizoma (0.0110), Magnoliae Cortex (0.0085), Notoginseng Radix Et Rhizoma (0.0075), Fritillariae Cirrhosae Bulbus (0.0057), Saposhnikoviae Radix (0.0055), Orobanchis Herba (0.0053), Polygoni Cuspidati Rhizoma et Radix (0.0050), Cremastrae Tuber (0.0050), Zanthoxyli Pericarpium (0.0049), and Capsici Fructus (0.0049) (Table 1). All ten candidates showed statistically significant overlap with hepatic cirrhosis–related proteins (*p*-values ranging from 6.38 × 10^−5^ to 0.019) and enrichment values between 3.88 and 6.79, corroborating the capacity of the multiscale diffusion-based approach to recover biologically meaningful herb–disease relationships.

Among the top-ranked candidates, prior literature support for hepatoprotective or antifibrotic activity was available for Magnoliae Cortex, Notoginseng Radix Et Rhizoma, Polygoni Cuspidati Rhizoma et Radix, and Capsici Fructus (Table 1). In contrast, several high-ranking herbs—most notably Cynanchi Atrati Radix Et Rhizoma, Saposhnikoviae Radix, Fritillariae Cirrhosae Bulbus, Orobanchis Herba, and Cremastrae Tuber—lacked curated evidence linking them to hepatic cirrhosis despite displaying significant protein overlap and enrichment, thereby nominating them as underexplored candidates warranting further mechanistic characterization and experimental validation.

### 3.2. Network Visualization and GSEA of Herbal Candidates for Hepatic Cirrhosis

**Figure 2:**
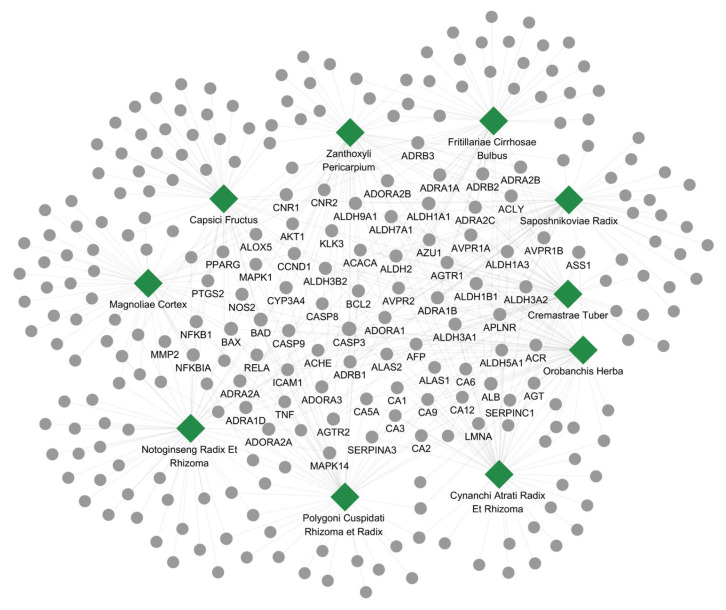
Herb–target interaction network of the top 10 candidate herbs with the highest correlation scores for hepatic cirrhosis. Green diamond represents an individual herb, while gray circles indicate the corresponding protein targets. Edge connections depict herb–target associations derived from curated interaction databases. Node size (both diamonds and circles) is proportional to the interaction frequency between each herb and its targets.

An interaction network connecting the 10 prioritized herbs to their protein targets was constructed and visualized (Figure 2). The resulting graph comprised 10 herb nodes, 261 protein-target nodes, and 500 herb–target edges, illustrating the characteristic multi-component, multi-target architecture of herbal therapeutics and their capacity to perturb the complex molecular landscape underlying cirrhosis. Of all mapped targets, 74 proteins were shared by three or more herbs, defining a focused subset of putative core effectors potentially representing convergent therapeutic mechanisms.

Functional inspection of these shared targets revealed enrichment along two principal axes pertinent to hepatic cirrhosis pathobiology. An inflammation and immune-regulatory axis was defined by hub proteins such as NFKB1, RELA, TNF, PTGS2, NOS2, AKT1, and MAPK1, indicating convergence on NF-κB and MAPK signaling cascades and their downstream inflammatory effectors (Figure 2). A parallel cell-death regulatory axis emerged from apoptosis-associated hubs including CASP3, CASP8, CASP9, BCL2, BAX, and BAD, pointing to potential modulation of both intrinsic and extrinsic apoptotic programs. The co-occurrence of these two functional clusters among the core shared targets suggests that the prioritized herbal candidates may confer therapeutic benefit in cirrhosis through the coordinated regulation of inflammatory signaling and programmed cell death.

### 3.3. Gene Ontology Enrichment Analysis of Key Protein Targets

To further interrogate the core protein targets of the top-ranked herbs, we performed KEGG pathway enrichment analysis (Table 2). The top enriched pathways prominently featured inflammatory and innate-immune signaling, including the TNF signaling pathway, Toll-like receptor signaling pathway, and NF-κB signaling pathway, together with an apoptosis-related pathway. These enrichments were driven by shared targets such as NFKB1, RELA, TNF, NFKBIA, IL6, PTGS2, NOS2, and CASP3/8/9. Additional pathways related to stress responses and survival signaling—including VEGF signaling, HIF-1 signaling, MAPK signaling, and PI3K–Akt signaling—were also significantly represented among the core targets.

**Figure 3:**
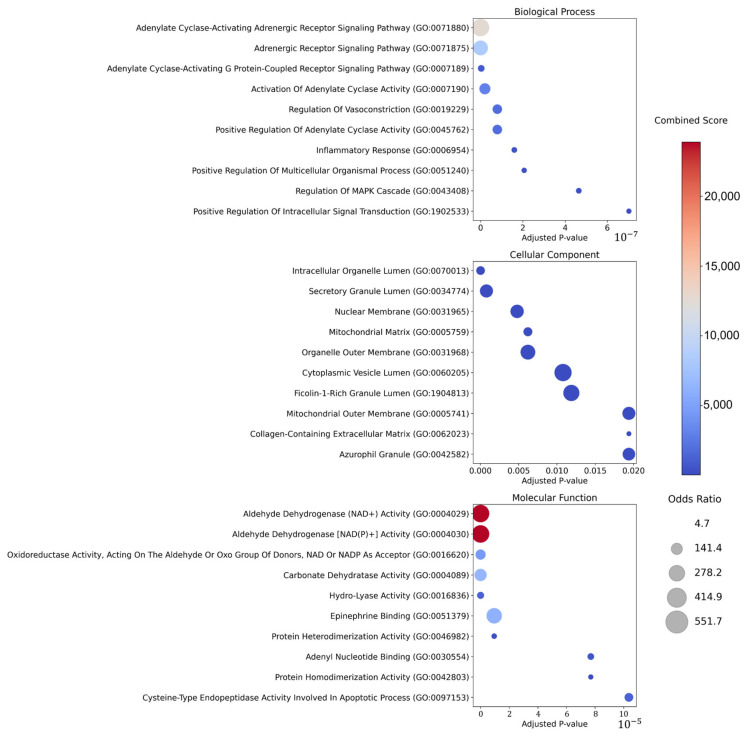
Gene Ontology (GO) enrichment analysis of core protein targets shared by prioritized herbal candidates in hepatic cirrhosis. The top 10 GO terms (by adjusted *p*-value) are shown for Biological Process (BP), Cellular Component (CC), and Molecular Function (MF). Each bubble represents a GO term; bubble size reflects the odds ratio (enrichment strength), and bubble color reflects the combined score. The x-axis indicates the *p*-value.

GO enrichment analysis was then performed on proteins commonly targeted by at least two of the 10 prioritized herbs using the GO Biological Process (BP), Cellular Component (CC), and Molecular Function (MF) databases, and the top 10 GO terms (ranked by adjusted *p*-value) were visualized for each category (Figure 3). In BP, enriched terms were mainly related to receptor- and second-messenger signaling and downstream inflammatory signaling, including adenylate cyclase–activating adrenergic/GPCR signaling, activation/positive regulation of adenylate cyclase activity, regulation of vasoconstriction, inflammatory response, and regulation of the MAPK cascade. In CC, enriched terms indicated localization to organelle and vesicle compartments (e.g., intracellular organelle lumen, secretory granule lumen, cytoplasmic vesicle lumen), mitochondrial compartments (mitochondrial matrix and outer membrane), and extracellular matrix–associated structures (collagen-containing extracellular matrix). In MF, enriched terms included enzyme activities related to redox and intermediary metabolism (e.g., aldehyde dehydrogenase activity and oxidoreductase activity), carbonate dehydratase activity, epinephrine binding, dimerization activities, and cysteine-type endopeptidase activity involved in apoptotic processes. Collectively, these GO results provide functional context for the shared targets of the prioritized herbs by highlighting signaling, inflammatory response, subcellular localization patterns, and enzymatic functions represented among the core proteins.

### 3.4. Analysis of Key Active Compounds in Selected Herbal Candidates

To investigate active ingredients from candidate herbs without reported evidence, we performed a compound-level investigation using the same multiscale network framework. For each herb, compound–target annotations were mapped onto the network and a biased random walk was applied to compute compound diffusion profiles. Compounds were then ranked by their correlation with the hepatic cirrhosis disease profile, and candidates showing both high correlation and significant target overlap were selected as putative active compounds (Table 3). This screening nominated succinic acid for Cynanchi Atrati Radix, Orobanchis Herba, and Cremastrae Tuber; fatty-acid–related compounds for Fritillariae Cirrhosae (e.g., 9-octadecenoic acid, octadecanoic acid, and oleic acid); bergapten for Saposhnikoviae Radix; and multiple sanshool-related constituents for Zanthoxyli Pericarpium. We then surveyed reported evidence at the compound level and observed prior support for succinic acid and the fatty-acid–related candidates, whereas bergapten and the sanshool-related constituents lacked curated evidence in this disease context, suggesting underexplored candidate actives for further validation.

**Figure 4:**
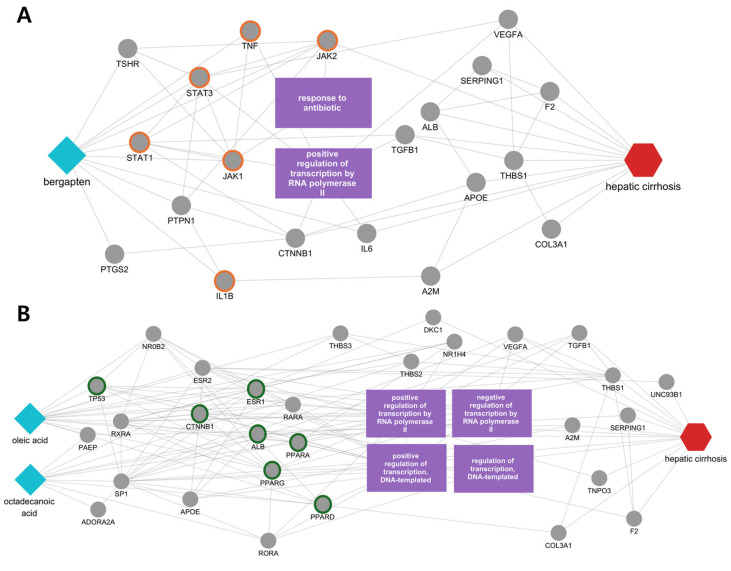
Disease-focused subnetworks in hepatic cirrhosis for candidate compounds lacking reported evidence in the curated screening. (**A**) bergapten (Saposhnikoviae Radix); (**B**) oleic acid and octadecanoic acid (Fritillariae Cirrhosae Bulbus). Cyan diamonds denote compounds, gray circles denote protein targets, purple rectangles indicate enriched biological functions, and the red hexagon denotes the disease. Outlined nodes highlight key proteins emphasized in the diffusion profiles. Orange-outlined proteins are associated with the JAK–STAT and TNF signaling pathways, while green-outlined proteins are associated with the PI3K–Akt and HIF-1 signaling pathways.

We then focused on candidate compounds with high correlation scores whose reported evidence was not available in our curated screening. Additional multiscale network analyses were conducted to generate mechanistic hypotheses. As a representative example, we visualized the multiscale network mechanism of bergapten (Saposhnikoviae Radix) in the context of hepatic cirrhosis (Figure 4A). In the bergapten-focused subnetwork, bergapten connected to multiple inflammation- and cytokine-related proteins, and the most prominent links were concentrated on proteins involved in JAK–STAT and TNF signaling, including STAT1, STAT3, IL1B, JAK1, TNF, and JAK2 (Figure 4A). These nodes were further connected to additional cirrhosis-relevant proteins (e.g., IL6, TGFB1, VEGFA, ALB, APOE, THBS1, F2, SERPING1, and COL3A1) and enriched biological functions (e.g., response to antibiotic; positive regulation of transcription by RNA polymerase II), providing a disease-contextualized view of how bergapten may perturb inflammatory signaling and downstream transcriptional programs.

**Figure 5:**
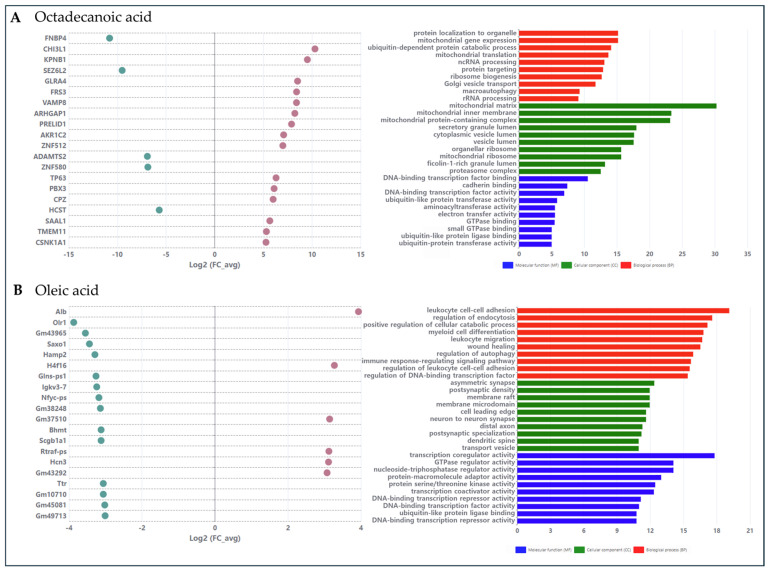
Public transcriptome analysis of two active compounds. (**A**) Octadecanoic acid; (**B**) Oleic acid. For each compound, the left panel shows representative DEGs ranked by their fold change. Pink dots indicate up-regulated genes, while green dots indicate down-regulated genes. The right panel shows GO enrichment of DEGs (BP/CC/MF).

To provide complementary transcriptome-level context for candidate active compounds, we analyzed publicly available gene expression datasets for octadecanoic acid and oleic acid (Figure 5). Differentially expressed genes (DEGs) were identified (left panels), and GO enrichment was performed using the DEG lists (right panels). For octadecanoic acid, GO terms were mainly related to mitochondrial/proteostasis and autophagy-associated processes, together with enzyme activities such as aldehyde dehydrogenase/oxidoreductase. For oleic acid, enriched GO terms highlighted immune/leukocyte-related processes, wound-healing–related processes, and regulatory functions including transcriptional and GTPase regulator activities. These transcriptome-level observations are consistent with the network-predicted mechanisms described above and provide independent gene expression evidence supporting the potential involvement of these fatty-acid compounds in hepatic cirrhosis–related biological processes.

### 3.5. In Vitro Screening of SR and FCB on LX-2 Cells

**Figure 6:**
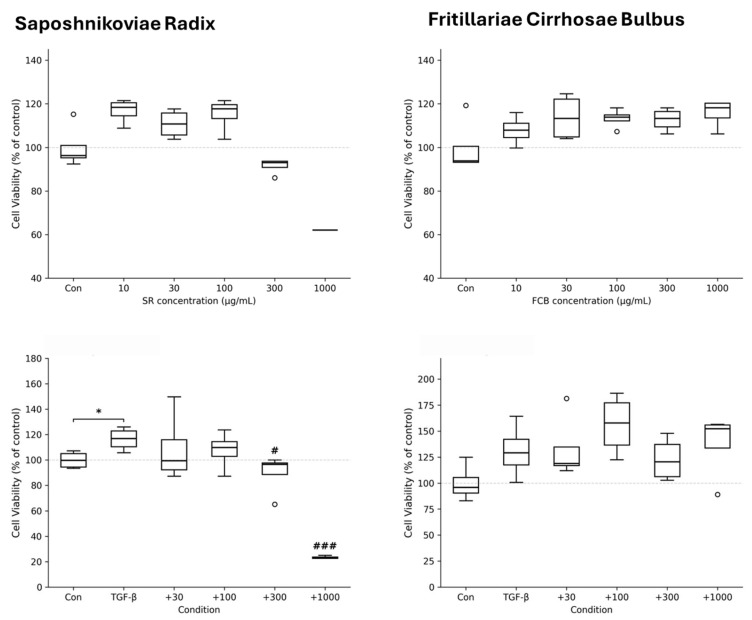
Effects of Saposhnikoviae Radix and Fritillariae Cirrhosae Bulbus on LX-2 hepatic stellate cell viability. * *p* < 0.05 vs. control; # *p* < 0.05, ### *p* < 0.001 vs. TGF-β1 alone.

To validate the computational predictions for liver cirrhosis, in vitro screening was performed using LX-2 hepatic stellate cells (Figure 6). SR showed no cytotoxicity up to 300 μg/mL, while 1000 μg/mL markedly reduced viability, indicating overt toxicity at this dose. FCB exhibited no cytotoxicity at any concentration tested. In the TGF-β1-stimulated model, TGF-β1 significantly increased cell viability to ~116% (*p* < 0.05 vs. control), reflecting stellate cell activation. SR co-treatment attenuated this response in a dose-dependent manner, with significant suppression at 300 μg/mL (*p* < 0.05 vs. TGF-β1) and 1000 μg/mL (*p* < 0.001 vs. TGF-β1), although the latter likely reflects cytotoxicity rather than a specific anti-fibrotic effect. FCB did not suppress TGF-β1-induced activation at any concentration. These findings suggest that SR, but not FCB, possesses anti-fibrotic potential within a non-cytotoxic range, partially supporting the network pharmacology predictions.

## 4. Discussion

In this study, we used a multiscale interactome framework to prioritize candidate herbs and active compounds for hepatic cirrhosis. By integrating curated herb–compound–target data with cirrhosis-associated proteins in a multiscale interactome and applying biased random-walk diffusion profiling, we identified a ranked set of candidate herbs, including Cynanchi Atrati Radix Et Rhizoma, Magnoliae Cortex, Notoginseng Radix Et Rhizoma, Fritillariae Cirrhosae Bulbus, and Saposhnikoviae Radix. Network and enrichment analyses of shared targets highlighted pathways and proteins related to inflammatory and innate-immune signaling and apoptosis regulation, supporting the biological plausibility of the prioritized candidates. In addition, compound-level prioritization nominated multiple putative active ingredients, and follow-up multiscale subnetwork analyses provided disease-contextualized mechanistic hypotheses for evidence-lacking candidates such as bergapten (Saposhnikoviae Radix) and fatty-acid–related compounds from Fritillariae Cirrhosae Bulbus. Together, these findings extend network-based herbal prioritization to hepatic cirrhosis and provide a structured set of candidate herbs and compounds for subsequent validation and therapeutic development.

The candidate herbs prioritized in this study were partly consistent with previously reported liver-related evidence, supporting the validity of the multiscale diffusion-profile approach. In particular, Magnoliae Cortex [25], Notoginseng Radix et Rhizoma [26], Polygoni Cuspidati Rhizoma et Radix [27], and Capsici Fructus [29,30] ranked highly in the cirrhosis-related network, suggesting that the model can recover herbs with existing literature support when herb–compound–target relationships are evaluated in a system-level interactome context. In contrast, several high-ranking herbs—Cynanchi Atrati Radix, Fritillariae Cirrhosae Bulbus, Saposhnikoviae Radix, Orobanchis Herba, Cremastrae Tuber, and Zanthoxyli Pericarpium—showed strong network association but lacked curated herb-level reported evidence in our screening, indicating that they may represent underexplored candidates for hepatic cirrhosis. Extending the analysis to the compound level, we nominated multiple putative actives from these evidence-lacking herbs, including succinic acid [31,32]. In contrast, several candidates—most notably bergapten (Saposhnikoviae Radix), octadecanoic acid and oleic acid (Fritillariae Cirrhosae Bulbus), and sanshool-related constituents from Zanthoxyli Pericarpium—had no reported evidence in the curated set and may represent novel bioactive leads for follow-up validation.

Across the multiscale network results, Saposhnikoviae Radix and Fritillariae Cirrhosae Bulbus shared two prominent pathway contexts—HIF-1 signaling and TNF signaling—suggesting common links to hypoxia-associated remodeling and chronic inflammatory injury in hepatic cirrhosis. Progressive fibrosis is accompanied by intrahepatic hypoxia, where HIF-1–related programs can couple inflammation to angiogenic and remodeling responses; thus, recurrent nodes such as VEGFA/IL6/STAT3 in the shared network can be interpreted in the context of hypoxia-responsive signaling during fibrogenesis [34,35]. In parallel, TNF signaling is centrally involved in sustained inflammatory stress and cell-death regulation, consistent with the shared presence of TNF/IL1B and apoptotic mediators such as CASP3 within the prioritized target space [36]. Notably, the two herbs showed partly different patterns of connectivity in the multiscale network. Saposhnikoviae Radix (bergapten) was more strongly connected to cytokine- and inflammation-related nodes within the JAK–STAT/TNF signaling context, consistent with reports that bergapten can suppress LPS-induced inflammatory cytokines with involvement of JAK/STAT signaling [37]. By comparison, Fritillariae Cirrhosae Bulbus (oleic acid and octadecanoic/stearic acid candidates) showed relatively stronger connections to PI3K–Akt/HIF-1–related survival and transcription-associated nodes, in line with literature indicating that fatty-acid exposure can reshape hepatocyte metabolic stress responses and organelle interactions, and that PI3K/Akt signaling is implicated in chronic liver inflammation/fibrosis and repair-associated phenotypes [38,39].

## Figures and Tables

**Table 1:** Top 10 ranked herbs by correlation with the hepatic cirrhosis diffusion profile.

Herb Name (Latin)	Correlation Score ^†^	Overlap (*p*-Value ^#^)	Enrichment	References (PMID)
Cynanchi Atrati Radix Et Rhizoma	0.0110	6/53 (0.0007)	5.49	-
Magnoliae Cortex	0.0085	7/50 (6.38 × 10^−5^)	6.79	32776713 [25]
Notoginseng Radix Et Rhizoma	0.0075	5/50 (0.0034)	4.85	15698847 [26]
Fritillariae Cirrhosae Bulbus	0.0057	5/50 (0.0034)	4.85	-
Saposhnikoviae Radix	0.0055	4/50 (0.019)	3.88	-
Orobanchis Herba	0.0053	4/50 (0.019)	3.88	-
Polygoni Cuspidati Rhizoma et Radix	0.0050	6/50 (0.0005)	5.82	23262250 [27]; 20607498 [28]
Cremastrae Tuber	0.0050	4/50 (0.019)	3.88	-
Zanthoxyli Pericarpium	0.0049	4/50 (0.019)	3.88	-
Capsici Fructus	0.0049	4/50 (0.019)	3.88	25289759 [29]; 27991776 [30]

^†^ The correlation score denotes the correlation score between the disease and herb diffusion profiles. The ^#^ symbol next to the *p*-value indicates values obtained using the hypergeometric test, applied to evaluate the significance of overlap between datasets.

**Table 2:** KEGG Pathway Enrichment Analysis of Shared Target Proteins from Hepatic Cirrhosis–Related Herbs.

Term	Overlap (*p*-Value)	Odds Ratio	Combined Score	Target Proteins
TNF signaling pathway	12/112 (8.49 × 10^−14^)	41.75	1389.6	NFKBIA, IL6, MAPK8, CASP8, CASP3, MAPK1, MAPK14, PTGS2, TNF, RELA, NFKB1, ICAM1
Apoptosis	13/142 (4.99 × 10^−14^)	35.63	1209.22	BAD, TNF, RELA, NFKB1, NFKBIA, CASP9, MAPK8, CASP8, CASP3, LMNA, BCL2, BAX, MAPK1
Toll-like receptor signaling pathway	9/104 (5.48 × 10^−10^)	31.32	726.09	NFKBIA, IL6, MAPK8, CASP8, MAPK1, MAPK14, TNF, RELA, NFKB1
NF-kappa B signaling pathway	7/104 (2.80 × 10^−7^)	23.09	380.53	NFKBIA, BCL2, PTGS2, TNF, RELA, NFKB1, ICAM1
VEGF signaling pathway	5/59 (5.31 × 10^−6^)	28.76	379.96	CASP9, BAD, MAPK1, PTGS2, MAPK14
HIF-1 signaling pathway	7/109 (3.67 × 10^−7^)	21.95	354.66	IL6, NOS2, BCL2, HMOX1, MAPK1, RELA, NFKB1
MAPK signaling pathway	7/294 (1.66 × 10^−4^)	7.73	74.12	MAPK8, CASP3, MAPK1, MAPK14, TNF, RELA, NFKB1
PI3K-Akt signaling pathway	7/354 (4.80 × 10^−4^)	6.37	53.79	CASP9, IL6, BAD, BCL2, MAPK1, RELA, NFKB1
TNF signaling pathway	12/112 (8.49 × 10^−14^)	41.75	1389.6	NFKBIA, IL6, MAPK8, CASP8, CASP3, MAPK1, MAPK14, PTGS2, TNF, RELA, NFKB1, ICAM1

**Table 3:** Putative active compounds from six candidate herbs lacking herb-level evidence and their association with hepatic cirrhosis.

Herb Name (Latin)	Compound (PubChem CID)	Correlation	Overlap (*p*-Value)	Enrichment	Reported Evidence (PMID)
Cynanchi Atrati Radix	succinic acid (1110)	0.0101	9/230 (0.0067)	2.17	29366478 [31]; 30186230 [32]; 37976628 [33]
Fritillariae Cirrhosae	Octadecanoic acid (5281)	0.0065	2/24 (0.0481)	1.32	-
	Oleic Acid (445639)	0.0064	5/127 (0.0392)	1.41	-
Saposhnikoviae Radix	bergapten (2355)	0.0044	2/15 (0.0199)	1.7	-
Orobanchis Herba	succinic acid (1110)	0.0101	9/230 (0.0067)	2.17	29366478 [31]; 30186230 [32]; 37976628 [33]
Cremastrae Tuber	succinic acid (1110)	0.0101	9/230 (0.0067)	2.17	29366478 [31]; 30186230 [32]; 37976628 [33]
Zanthoxyli Pericarpium	hydroxy-γ-isosanshool (14135316)	0.022	2/2 (0.0002)	3.67	-
	tetrahydrobungeanool (5321844)	0.022	2/2 (0.0002)	3.67	-
	hydroxy-β-sanshool (10220912)	0.022	2/2 (0.0002)	3.67	-
	hydroxy-γ-sanshool (14135317)	0.022	2/2 (0.0002)	3.67	-
	hydroxy-α-sanshool (10084135)	0.013	2/4 (0.0013)	2.9	-

## Data Availability

The datasets supporting the conclusions of this article are included within the article.
